# Comparing Response Evaluation Methods for PRRT in Neuroendocrine Tumors: Insights from an Exploratory Study

**DOI:** 10.3390/cancers18081267

**Published:** 2026-04-16

**Authors:** Priscilla Guglielmo, Carlo Carnaghi, Alexia Francesca Bertuzzi, Alice Laffi, Sara Damiani, Ugo Carlo Riva, Michela Olivieri, Manuela Marenco, Laura Evangelista

**Affiliations:** 1 Department of Biomedical Sciences, Humanitas University, Pieve Emanuele, 20072 Milan, Italy; 2 Nuclear Medicine Unit, Humanitas Gavazzeni, 24125 Bergamo, Italy; 3 Medical Oncology Unit, Humanitas Istituto Clinico Catanese, Misterbianco, 95045 Catania, Italy; 4 Medical Oncology and Haematology Unit, Humanitas Cancer Center, IRCCS Humanitas Research Hospital, Rozzano, 20089 Milan, Italy; 5 Division of Medical Oncology, Humanitas Gavazzeni, 24125 Bergamo, Italy; 6 Medical Physics Unit, IRCCS Humanitas Research Hospital, Rozzano, 20089 Milan, Italy; 7 Nuclear Medicine Unit, IRCCS Humanitas Research Hospital, Rozzano, 20089 Milan, Italy

**Keywords:** NET, PRRT, neuroendocrine tumors, radioligand therapy, response evaluation

## Abstract

**Background/Objectives**: Assessing treatment response in neuroendocrine tumors (NETs) remains challenging. The multifactorial mechanism of action of peptide receptor radionuclide therapy (PRRT) further complicates response evaluation, particularly in the absence of standardized criteria. This study aimed to compare different imaging-based response assessment methods after PRRT and to explore their relationship with clinical outcomes, particularly progression-free survival (PFS). **Methods**: In this single-center retrospective study, we analyzed NET patients treated with PRRT between 2020 and 2024 who underwent [^68^Ga]Ga-DOTATOC PET/CT before and after therapy, with a minimum follow-up of 12 months. Five response criteria were evaluated: (a) Krenning Score changes; (b) adapted PERCIST criteria (MORE); (c) ZP-normalized parameter; (d) qualitative visual PET assessment; and (e) RECIST-based morphological response on contrast-enhanced CT. Imaging findings were correlated with clinical outcomes. Nonparametric analyses were performed using the MedCalc^®^ software. **Results**: Thirty-one patients (median age 63 years; 17 males) with NET were evaluated after PRRT. Post-PRRT PET/CT was performed at a median of 3 months. Response rates varied across methods, with higher rates noted using functional imaging (MORE 66%, Visual PET 68%, ZP 70%) compared to RECIST (40%) and Krenning score (21%). After a median follow-up of 37 months, 58% of patients experienced disease progression. Although no significant association was found between the response criteria and progression (*p* > 0.05), functional imaging showed a trend toward better correlation with longer progression-free survival. **Conclusions**: Functional PET-based responsecriteria suggest association with progression-free survival compared to RECIST criteria and Krenning score changes. However, given the exploratory nature of the study and its methodological limitations, these observations should be considered hypothesis-generating only. They do not provide definitive evidence of superiority over established assessment methods and therefore should not be interpreted as practice-changing.

## Simple Summary

Evaluating how patients respond to peptide receptor radionuclide therapy (PRRT) for neuroendocrine tumors (NETs) remains challenging, as standardized criteria are lacking and different imaging approaches may provide discordant results. This study compared different imaging-based approaches, including the PET and CT criteria, in 31 patients to explore their relationship with clinical outcomes. We observed substantial variability in response classification across the criteria. In patients with early progression, some PET-based approaches more frequently classified patients as responders, whereas in those with longer disease control, metabolic parameters such as the ZP index and qualitative PET assessment were associated with delayed progression. Overall, these findings suggest that functional imaging may provide complementary information to conventional CT; however, results should be considered exploratory and require validation in larger cohorts.

## 1. Introduction

Neuroendocrine tumors (NETs) are rare neoplasms that originate from endocrine or neuroendocrine cells [1]. Over recent decades, the incidence of NETs has increased, with gastroenteropancreatic (GEP) subtypes representing the most frequent entities [2]. Five-year survival rates in NET patients vary greatly, as the are primarily determined by tumor site, histological grade, proliferative activity, metastatic burden at presentation, and therapeutic options [1]. Management and therapy of NETs are influenced by disease grade (expressed as the Ki67 index and mitotic count [2]), stage, and underlying pathology. To date, surgical resection remains the first-line treatment for localized disease; other options include long-acting somatostatin analogues (SSAs), whereas second-line options include peptide radioreceptor therapy (PRRT) and targeted agents (e.g., mTOR inhibitors, TKIs) which are commonly used as next-line therapies [3]. Chemotherapy, in particular the combination of capecitabine and temozolomide, may be considered in selected patients [4]. In particular, PRRT using somatostatin receptor-ligand analogues, such as [^177^Lu]Luoctreotate (^177^Lu-PRRT) or [^90^Y]Y-octreotate (^90^Y-PRRT), has proven to be an effective systemic treatment for unresectable or metastatic NETs that have progressed on first-line therapy with SSAs [5], as demonstrated by the NETTER-1 trial [6], which led to the approval of [^177^Lu]Lu-DOTATATE by the Food and Drug Administration (FDA) in 2018. Eligibility for PRRT is based on the degree of somatostatin receptor (SSTR) expression on imaging, which is most frequently evaluated using the Krenning Score (KS), originally designed for evaluating [^111^In]-pentetreotide uptake [7], as a 5-point scale comparing the intensity of tracer uptake within a lesions to that of the liver and spleen.

Nevertheless, the clinical benefits of PRRT remain heterogeneous and often unpredictable [8], and many studies have investigated the determinants of treatment failure or suboptimal outcomes in patients exhibiting somatostatin-avid lesions on [^68^Ga]Ga-DOTATATE PET/CT [9,10,11]. Prognostic determinants, including tumor grade, the Ki67 index, intra- and inter-lesional variability in SSTR expression [12,13,14], and the presence of [^18^F]FDG-avid metastatic deposits [15], demonstrate comparable predictive value for PRRT efficacy; nevertheless, their capacity to reliably stratify patient outcomes remains limited.

An accurate tumor response assessment is crucial, considering that the recognition of disease relapse or progression facilitates the immediate initiation of optimal therapeutic interventions. NET typically exhibits rich vascularization, and PRRT may induce alterations not only in lesion dimensions but also in their radiological enhancement characteristics and attenuation profiles [16]. Therefore, relying solely on dimensional variations observed on CT imaging, as defined by the RECIST (Response Evaluation Criteria in Solid Tumors) criteria, is insufficient for a comprehensive evaluation of therapeutic response.

In the Delphic consensus on GEP-NET [16], both the RECIST 1.1 criteria [17] and functional imaging metrics from PET were considered. For this latter imaging modality, standardized uptake value (SUV) is often used, although it is considered inadequate due to pronounced variability in somatostatin receptor expression and the diverse histopathological features underpinning tumor heterogeneity.

Wahl et al. [18] introduced in 2009 the Positron Emission Tomography Response Criteria in Solid Tumors (PERCIST) as a framework for assessing therapeutic efficacy in solid malignancies by quantifying metabolic activity via SUV.

PERCIST has demonstrated the highest inter-observer concordance and identified a greater proportion of non-responders to PRRT compared with RECIST 1.1 and modified RECIST, indicating superior performance in evaluating response to treatment in NETs [19]. Such capability holds promise in contexts where early response determination is critical, including clinical trial settings. Nonetheless, its implementation is limited by the necessity for baseline functional imaging, which is frequently unavailable [20].

The aim of our study is to compare different imaging-based response assessment methods after PRRT and explore their relationship with clinical outcomes, particularly progression-free survival (PFS).

## 2. Materials and Methods

**Figure 1:**
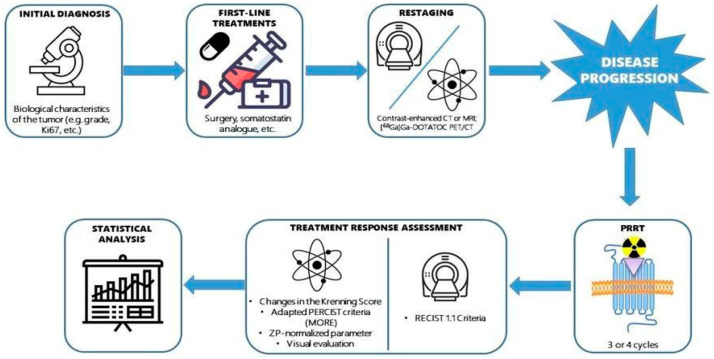
Study design.

Figure 1 describes the study design of our analysis.

### 2.1. Patients Population

In this retrospective single-center study, we selected from an internal registry those patients who fulfilled the following inclusion criteria: (1) subjects with grade 1 or 2 NET who exhibited disease progression after first-line treatments and subsequently received PRRT, after evaluation by a multidisciplinary tumor board and in accordance with Italian healthcare reimbursement specifications, at the Nuclear Medicine Department of IRCCS Humanitas Hospital (Rozzano, Italy) between 2020 and 2024; (2) patients who underwent [^68^Ga]Ga-DOTATOC PET/CT immediately before and after PRRT; (3) a minimum follow-up period of 12 months after the post-PRRT [^68^Ga]Ga-DOTATOC PET/CT examination; (4) age > 18 years old; (5) patients able to provide written informed consent or whose consent was provided by legal representatives. Availability of ceCT imaging was also recorded.

The exclusion criteria were: (a) incomplete [^68^Ga]Ga-DOTATOC PET/CT data, (b) suboptimal quality of PET/CT images due to partial extravasation of [^68^Ga]Ga-DOTATOC from the injection site, and (c) participation in clinical trials. For all patients, we collected demographics, surgical information (if available), histology, PRRT treatment schedule, and follow-up data. Clinical outcome (relapse vs no evidence of progressive disease) was used for survival analyses. The institutional Ethics Committee approved the study (approval number 357/25, date of approval 18 June 2025).

### 2.2. [^68^Ga]Ga-DOTATOC PET/CT Acquisition

SSTR-based PET/CT imaging was performed at least four weeks after the most recent SSA administration. Whole-body [^68^Ga]Ga-DOTATOC PET scans were performed approximately 60 min after the intravenous administration of [^68^Ga]Ga-DOTATOC (2.2–2.5 MBq/kg) with patients in the supine position, in accordance to European Association of Nuclear Medicine (EANM) guidelines [19]. Imaging was conducted using Siemens Biograph 6 LSO, Siemens Vision 600 (Siemens, Erlangen, Germany), or General Electric Discovery 690 (GE Healthcare, Waukesha, WI, USA). Both scanners were accredited under the EANM Research Ltd. (EARL, Vienna, Austria) quality assurance program. The acquisition protocol was as follows: first, a low-dose whole body CT scan was performed for anatomical localization (kVp = 120, Auto mA-30–150 mA); then, a whole-body PET study (1.5–2 min/FOV) was acquired.

Both qualitative visual assessments and semiquantitative analyses were carried out at baseline and post-PRRT evaluations using a dedicated PET/CT workstation (Advantage Workstation 4.6, GE Healthcare, Milwaukee, WI, USA) with PET-Volume Computer-Assisted Reading (PET-VCAR) software (GE Healthcare, Milwaukee, WI, USA).

### 2.3. Radiopeptide Treatment (PRRT)

Patients underwent administration of [^177^Lu]Lu-DOTATATE through a controlled intravenous infusion lasting approximately 10–15 min. All subjects received renal prophylaxis consisting of 1500 mL of an amino acid solution (250 mL NaCl supplemented with 5% lysine hydrochloride and 250 mL of 10% L-arginine hydrochloride) delivered over a 4-h period. Each cycle (maximum dosage: 7.4 GBq) was administered every 8 weeks, for a maximum of 4 cycles.

### 2.4. Methods of Treatment Response Assessment

The evaluation of [^68^Ga]Ga-DOTATOC PET/CT images was conducted by two expert nuclear medicine physicians (P.G. and L.E.) with more than 5 years of experience in NET imaging. Patients were classified as “responders” in cases of complete response (CR) or partial response (PR), whereas they were classified as “non-responders” in cases of stable disease (SD) or progression disease (PD), according to each evaluation method described below. Table 1 summarizes the selected response criteria.

#### 2.4.1. Change in the Krenning Score (KS)

The KS [7] of the lesion with the highest uptake for each patient was evaluated, and changes in the KS between pre- and post-PRRT [^68^Ga]Ga-DOTATOC PET/CT were reported.

#### 2.4.2. “The Adapted” PERCIST Criteria (MORE)

The evaluation of SSTR density within tumor tissue and normal liver parenchyma was performed through a semiquantitative approach, employing both the maximum and mean SUV (named SUVmax and SUVmean, respectively). For each tumor site, as well as for normal liver, SUVmean was derived by applying a 42% isocontour threshold on transaxial, attenuation-corrected PET reconstructions. Lesion delineation on PET was guided by the corresponding anatomical margins defined on CT imaging; whenever the automatically generated isocontour extended beyond or fell short of the CT-defined boundaries, manual adjustment of the PET-derived lesion contour was performed. The average hepatic SUV was adopted as the reference parameter. Subsequently, the SUVmax measured in the neoplastic site was normalized to the hepatic SUVmean according to the following formula:

normalized uptake in tumor (SUVratio) = SUVmax Tumor/SUVmean liver

Response assessment using PET was conducted through the adaptation for [^68^Ga]Ga-DOTATOC PET/CT imaging, according to the guidelines proposed by the European Organization for Research and Treatment of Cancer (EORTC) [21], and in accordance with the methodology already described in a study published by Zwirtz et al. [22], termed molecular response evaluation (MORE). Disease status was determined based on SUVmax criteria: PD was defined as a >25% increase or the emergence of new lesions; SD as changes within −25% to +25% in target lesions; PR as a ≥25% reduction; and CR as the disappearance of all PET-detectable lesions.

#### 2.4.3. ZP-Normalized Parameter

Following the methodology described by Zwirtz et al. [22], we also calculated the quantitative hybrid parameter ZP, which derives from the linear energy transfer concept, directly related to tissue density (mass per unit volume). In CT, attenuation expressed in Hounsfield Units (HU) reflects this property. Radiation energy deposition depends on the number of β-particles, which in turn is proportional to both somatostatin receptor density and tissue composition. The following formulas were then applied:

ZP (Target) = SUVmean (Target) × HU (Target)

ZPnormalized (Target) = normalized SUVmean (Target) × HU (Target)

Unlike the aforementioned study, in which ZP was calculated only in patients undergoing simultaneous contrast-enhanced CT during SSTR PET/CT to avoid potential misregistration with separately acquired diagnostic CT, in our cohort ZP was derived from non–contrast-enhanced, coregistered CT images, since none of the patients received intravenous contrast simultaneously with the [^68^Ga]Ga-DOTATOC PET/CT. For consistent response assessment, we adopted a classification analogous to MORE for ZP parameter. PD was defined as an increase of ≥25% in the product of HU and SUVmean, while a decrease of ≥25% indicated PR. CR was assigned when no lesions were detectable on either CT or PET. Lesions not fulfilling these criteria were categorized as SD.

#### 2.4.4. Visual PET Analysis

Qualitative assessment of treatment response on PET imaging was based on visual evaluation of tracer uptake changes in known lesions, focusing on reduction, stability, or progression in comparison with baseline scans. Lesions were judged according to their relative intensity and extent of uptake with respect to physiological background activity.

#### 2.4.5. RECIST-Based Morphological Response

According to the current European Neuroendocrine Tumor Society recommendations [23], evaluation of therapeutic efficacy in radionuclide-based treatments was performed using RECIST 1.1 [17]. At baseline, target lesions were identified, and their longest diameters were measured, with a maximum of five lesions overall and no more than two per organ included in the assessment. The sum of these diameters provides the reference for subsequent evaluations. Response was then categorized into four main outcomes: CR, characterized by the disappearance of all target lesions; PR, defined as a reduction of at least 30% in the cumulative diameter compared with baseline; PD, determined by an increase of at least 20% in the sum of diameters or the appearance of new lesions; and SD, representing changes that do not meet the thresholds for either response or PD. Non-target lesions were also assessed qualitatively, and their unequivocal progression contributed to the definition of overall disease status.

### 2.5. Follow-Up Outcome

Disease progression was determined using a composite approach integrating morphologic imaging findings and clinical evaluation. Radiologic progression was assessed according to RECIST 1.1 [17], based on ceCT. Progression-free survival (PFS) was defined as the time interval between the end-of-treatment PET/CT examination and the date of documented disease progression or death from any cause, whichever occurred first. Patients who were alive and free of progression at the time of last follow-up were censored at that date. In addition to imaging-based criteria, clinical progression was considered when there was clear evidence of disease worsening not fully captured by RECIST measurements, including significant deterioration in disease-related symptoms, declines in performance status attributable to tumor burden, or the need for a change in systemic therapy due to suspected disease progression. When discrepancies arose between imaging findings and clinical assessment, progression was adjudicated by multidisciplinary consensus.

### 2.6. Statistical Analysis

The overall characteristics of the population were described using distribution tables and proportional measures. The correlations between continuous and categorical variables were assessed by using non-parametric tests. PFS was analyzed by Kaplan–Meier curves. A log-rank test (Mantel–Cox) was run to compare survival curves. *p*-values < 0.05 were considered statistically significant. MedCalc^®^ software for Windows, version 23.3.7 (MedCalc Software, Ostend, Belgium) was used.

## 3. Results

After screening the local database, 31 patients (M:F 17:14; median age 63 years, range 29–82) met the inclusion criteria. Table 2 summarizes patient characteristics.

Most patients (15/31, 48%) presented with small intestinal NETs, followed by pancreatic NETs in eleven cases (36%), three patients (10%) presented with a mesenteric mass, one with a cecal NET (3%), and one with a rectal NET (3%). Seven patients (23%) presented with syndromic disease, which comprised four patients with diarrhea due to carcinoid syndrome, one patient with Cushing’s syndrome, and two affected by carcinoid syndrome not otherwise detailed. PRRT was the second-line treatment in 18 patients (58%), the third-line treatment in 9 patients (29%), the fourth-line treatment in 3 (10%), and the eighth-line treatment in 1 (3%). All patients were previously treated with SSAs, 8 patients with additional Everolimus, 2 patients also with chemotherapy, and 4 patients with liver-directed local therapies.

### 3.1. [^68^Ga]Ga-DOTATOC PET/CT

[^68^Ga]Ga-DOTATOC PET/CT scans were performed before PRRT (baseline) and after its completion, with the post-treatment scan conducted at a median of 3 months (range: 1–8). In total, 76 lesions were assessed at baseline in 31 patients (range: 1–5 per patient): 42 located in the liver, 12 in lymph nodes, 9 mesenteric masses, 5 bone lesions, 3 in the small intestine, 2 pelvic masses, and one pancreatic lesion. Two lesions were classified as indeterminate due to overlap with adjacent structures. This was further complicated by the lack of contrast-enhanced imaging.

### 3.2. Treatment Response Assessment

Table 3 summarizes treatment responses according to each evaluation method. A substantial variability in response classification was observed across the different criteria. When patients were categorized as responders (CR + PR) and non-responders (SD + PD), the proportion of responders varied markedly depending on the method applied.

Based on the KS, 5 patients (21%) were classified as responders and 23 (79%) as non-responders. Using the MORE criteria, 19 out of 29 patients (66%) were classified as responders and 10 (34%) as non-responders. Similarly, with the ZP-normalized parameter, 19 patients (70%) were categorized as responders, and 8 (30%) as non-responders. Visual PET assessment identified 21 responders (68%) and 10 non-responders (32%). In contrast, according to RECIST 1.1, 8 patients (40%) were classified as responders and 12 (60%) as non-responders.

### 3.3. Progression-Free Survival

After a median follow-up of 37 months from the last PRRT cycle, 18 patients (58%) experienced disease progression, with a median time to progression of 20 months (range 3–47). Six patients (33%) experienced progression within 12 months after the completion of PRRT. Most of these case of PD (14/18, 78%) occurred in patients with a Grade 2 tumor at initial diagnosis, and predominantly in those with pancreatic NETs (8/18, 44%) or small intestinal NETs (8/18, 44%); in addition, this subgroup included all three deaths observed in the cohort, all of them NET-related. Based on RECIST criteria, 44% of non-responder patients experienced disease progression. Conversely, 25%, 29%, 19% and 81% with no response to PRRT, as assessed by MORE, Visual PET, ZP and KS criteria, respectively, experienced disease progression. Table 4 reports the number of patients categorized as responders or non-responders by MORE, ceCT, visual PET assessment, ZP parameter, and KS within each progression subgroup.

Across all response criteria, no statistically significant association with progression groups was observed (all *p* > 0.05). However, functional imaging methods (MORE, Visual PET, and ZP) consistently showed higher response rates, particularly in patients with longer progression (>20 months), where response reached 100%. In contrast, ceCT and KS demonstrated lower and more variable response rates, especially in patients without progression.

**Figure 2:**
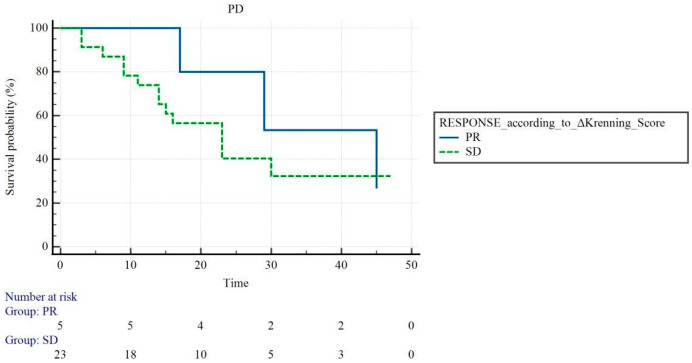
Kaplan–Meier curve for PFS according to Krenning score variation.

Interestingly, among functional criteria, only the response assessment based on KS showed a graphical trend with PFS, but it did not show a statistically significant association (*p* = 0.38) (Figure 2; *p* = 0.38).

## 4. Discussion

In this exploratory study, we compared multiple functional and morphological imaging criteria to assess responses to PRRT in patients with NETs, with the aim of exploring their relationship with clinical outcomes, particularly PFS. The main finding of this analysis is the marked variability in response classification across different evaluation methods, with functional PET-based approaches (MORE, visual PET, and ZP parameter) consistently identifying a higher proportion of responders compared with RECIST and KS. However, this variability did not translate into statistically significant differences in PFS, highlighting the limited predictive value of the current response criteria in this setting. Interestingly, a considerable proportion of patients classified as responders subsequently experienced disease progression. This observation underscores the limited ability of both functional and morphological criteria to reliably predict clinical outcomes, particularly in the early post-treatment phase.

Functional imaging approaches appeared to provide additional biological insight beyond size-based evaluation, as they captured changes in somatostatin receptor expression and tracer uptake. In patients with longer PFS (>20 months), all were classified as responders according to PET-based criteria, suggesting that a measurable functional response may reflect more favorable tumor biology. However, this finding must be interpreted cautiously. In patients experiencing early progression (<20 months), a comparable rate was reported for morphological and functional criteria. ceCT classified 75% of these patients as responders, whereas PET-based methods identified a higher proportion of responders (~60–70%), mainly driven by partial responses. Changes in SSTR expression may reflect tumor heterogeneity, including dedifferentiation and more aggressive disease phenotypes, rather than true therapeutic efficacy. Accordingly, dual-tracer imaging combining [^68^Ga]Ga-DOTA-peptides and [^18^F]FDG may offer complementary information, although this approach is not yet standardized and was beyond the scope of the present study [24,25]. In contrast, RECIST-based assessment showed lower response rates and substantial variability, likely reflecting its known limitations in NETs, where treatment effects may manifest as changes in lesion composition or vascularity rather than size reduction [17]. This limitation is particularly relevant in liver-dominant disease and heterogeneous lesions [26,27].

Our findings also highlight the limited role of the KS in post-treatment response assessments. The score classified most patients as non-responders across all progression groups, supporting the interpretation that it primarily reflects baseline receptor expression rather than dynamic treatment-induced changes. Interestingly, although a graphical trend with PFS was observed, no statistically significant association was found. This further supports the notion that the KS may be more appropriately considered a selection or prognostic tool, rather than a response evaluation method [28,29].

Another important aspect concerns the timing of post-treatment imaging. In our study, response was assessed after completion of PRRT, in line with current guidelines [5]. However, the high proportion of stable disease and discordance between response classification and outcomes suggest that early post-treatment imaging may not fully capture the extend of treatment effects. The optimal timing for response evaluation remains uncertain. Interim imaging may be confounded by pseudoprogression, particularly on morphological imaging, due to treatment-related inflammation or necrosis [22,23]. Indeed, lesions classified as stable or mildly progressive shortly after treatment may subsequently demonstrate partial response at later time points [6]. Therefore, careful interpretation of early imaging findings is required, and longitudinal assessment may provide a more accurate evaluation of treatment effects [22,26].

Taken together, our findings emphasize the complexity of response assessment in PRRT-treated NETs. No single imaging modality appears sufficient to fully characterize treatment response. Functional and morphological criteria provide complementary but incomplete information, and their interpretation requires integration with the clinical context. These results support the need for more comprehensive and biologically meaningful response frameworks, potentially incorporating: (1) hybrid imaging parameters; (2) longitudinal imaging evaluation, and (3) clinical and biochemical markers. Such approaches may better reflect tumor heterogeneity and treatment effects.

This study has several methodological limitations that should be explicitly acknowledged. First, the retrospective and single-center design introduces an inherent risk of selection bias and limits the generalizability of the findings. Patient inclusion was based on the availability of complete imaging and follow-up data, which may have resulted in a non-representative cohort. Second, the small sample size (*n* = 31) substantially limits statistical power. This increases the risk of type II errors and reduces the ability to detect significant associations between response criteria and clinical outcomes. Therefore, all findings should be interpreted as exploratory. Third, heterogeneity in data availability across response criteria led to variable sample sizes in the analyses (e.g., incomplete RECIST data), potentially introducing bias and limiting direct comparability between methods. Fourth, variability in imaging timing (post-PRRT PET/CT performed between 1 and 8 months) may have influenced response classification. Given the identified delayed biological effects of PRRT, early or non-uniform assessment may result in misclassification, including pseudoprogression or underestimation of response. Fifth, the definition of the primary endpoint (PFS) relied on a composite assessment incorporating both radiological and clinical criteria, including multidisciplinary judgment. While this reflects real-world practice, it introduces a degree of subjectivity and potential variability in outcome classification. Sixth, the use of non-contrast CT for ZP parameter calculation represents a methodological limitation, as attenuation values (HU) may be less reliable compared to contrast-enhanced imaging, potentially affecting the robustness and reproducibility of this hybrid metric. Seventh, none of the evaluated response criteria accounted for intra- and inter-lesional heterogeneity, which is a well-recognized feature of NETs and may significantly impact treatment response and imaging interpretation. Finally, the study did not include external validation or prospective confirmation, and no adjustment for potential confounders (e.g., tumor grade, prior therapies, tumor burden) was performed, further limiting the strength of causal inference.

## 5. Conclusions

In conclusion, although functional imaging methods identified a higher proportion of responders, none of the evaluated criteria showed a significant association with PFS, and earlyresponse classification did not reliably predict clinical outcomes. These findings reinforce that current response criteria in PRRT remain suboptimal and insufficiently standardized. Given the exploratory nature of this study, the results should be considered hypothesis-generating. Future prospective studies in larger cohorts are warranted to validate these findings and to develop integrated response models that better capture the biological complexity of NETs. We have now initiated a large national data collection to further investigate this issue.

## Figures and Tables

**Table 1:** Summary of the treatment response evaluation methods.

Treatment Response Methods	Complete Response	Partial Remission	Stable Disease	Progressive Disease
Change in the Krenning Score (KS)	KS value 0 or 1	Reduced KS value (KS > or =2).	Same KS value (KS > or =2).	Increased KS value
MORE (molecular response evaluation) Criteria	Complete disappearance of uptake in all lesions.	≥25% reduction in the sum of SUVmax after more than one cycle of treatment.	Does not meet the criteria for complete response (CR), partial response (PR), or progressive disease (PD).	≥25% increase in the sum of SUVmax or at least one new lesion.
ZP parameter	No lesions detectable in CT or PET.	≥25% reduction in the product of SUVmean and HU.	Does not meet the criteria for complete response (CR), partial response (PR), or progressive disease (PD).	≥25% increase in the product of SUVmean and HU.
Visual PET evaluation	No lesions detectable in CT or PET.	Visual persistence of comparable uptake pattern post-therapy.	Visual decrease in uptake intensity and/or disease burden.	Visual increase in uptake or extension of the lesions and/or appearance of new lesions.
RECIST 1.1	Disappearance of all lesions and no new lesions.	≥30% reduction in the sum of the greatest diameter and no new lesions.	Does not meet the criteria for complete response (CR), partial response (PR), or progressive disease (PD).	≥20% increase in the sum of the greatest diameters or at least one new lesion.

**Table 2:** Patient characteristics.

Patient Characteristics (*n* = 31)	
Age in years, median (range)	63 (29–82)
Sex, male: female ratio	17:14
Grade NET, *n* (%)	
Grade 1	11 (35)
Grade 2	20 (65)
Target lesions per patient, median (range)	2 (1–5)
Number of PRRT cycles (range)	4 (3–4)
Time between the first diagnosis and the first PRRT in months, median (range)	52 (8–330)
Duration of follow-up after last PRRT in months, median (range)	37 (5–54)
Duration of follow-up after last [^68^Ga]Ga-DOTATOC PET/CT in months, median (range)	33 (2–50)
Interval between PRRT * and first progression (months)	20 (3–47)

* Peptide Radionuclide Receptor Therapy.

**Table 3:** Distribution of responses for each evaluation method.

	Responders		Non-Responders		
Evaluation Method	CR (%)	PR (%)	SD (%)	PD (%)	Total (Missing Data or NE)
MORE Criteria	0	19 (66)	9 (31)	1 (3)	29 (2)
ZP Parameter	0	19 (70)	8 (30)	0	27 (4)
Change in the Krenning Score	0	5 (21)	23 (79)	0	28 (3)
Visual PET evaluation	0	21 (68)	8(26)	2 (6)	31 (0)
RECIST 1.1	0	8 (40)	10 (50)	2 (10)	20 (11)

CR = complete response; PR = partial response; SD = stable disease; PD = progressive disease; NE = not evaluable.

**Table 4:** Distribution of the progression in accordance with the response criteria.

	MORE			RECIST			Visual PET			ZP			Krenning Score		
	*n*	Resp (%)	Non- Resp (%)	*n*	Resp (%)	Non- Resp (%)	*n*	Resp (%)	Non- Resp (%)	*n*	Resp (%)	Non- Resp (%)	*n*	Resp (%)	Non- Resp (%)
Prog < 20 mo.	10	6 (60)	4 (40)	4	3 (75)	1 (25)	12	7 (58)	5 (42)	10	7 (70)	3 (30)	11	1 (9)	10 (91)
Prog > 20 mo.	6	6 (100)	0	5	2 (40)	3 (60)	6	6 (100)	0	6	6 (100)	0	5	2 (40)	3 (60)
No progression	13	7 (54)	6 (46)	11	3 (27)	8 (73)	13	8 (62)	5 (38)	11	6 (54)	5 (46)	12	2 (17)	10 (83)
*p*-value		*0.383*			*p *= *0.406*			*0.421*			*0.353*			*0.273*	

Legend: Resp = responders (CR + PR); Non-resp = non-responders (SD + PD); Prog = progression.

## Data Availability

The data presented in this study are available from the corresponding author upon reasonable request.
